# Regulation of Nuclear Import During Differentiation; The IMP α Gene Family and Spermatogenesis

**DOI:** 10.2174/138920207782446151

**Published:** 2007-08

**Authors:** J.E Holt, J.D Ly-Huynh, A Efthymiadis, G.R Hime, K.L Loveland, D.A Jans

**Affiliations:** 1 Monash University, Department of Biochemistry and Molecular Biology, Nuclear Signalling Laboratory, Clayton, Australia; 2 ARC Centre of Excellence in Biotechnology; 3 University of Melbourne, Department of Anatomy and Cell Biology, Parkville, VIC Australia; 4 Monash Institute for Medical Research, Clayton, VIC, Australia

**Keywords:** Importin alpha, promoter, spermatogenesis, nuclear transport.

## Abstract

Access to nuclear genes in eukaryotes is provided by members of the importin (IMP) superfamily of proteins, which are of α- or β-types, the best understood nuclear import pathway being mediated by a heterodimer of an IMP α and IMP β1. IMP α recognises specific targeting signals on cargo proteins, while IMP β1 mediates passage into, and release within, the nucleus by interacting with other components of the transport machinery, including the monomeric guanine nucleotide binding protein Ran. In this manner, hundreds of different proteins can be targeted specifically into the nucleus in a tightly regulated fashion. The IMP α gene family has expanded during evolution, with only a single IMP α (Srp1p) gene in budding yeast, and three (IMP α1, 2/pendulin and 3) and five (IMP α1, -2, -3, -4 and -6) IMP α genes in *Drosophila melanogaster* and mouse respectively, which fall into three phylogenetically distinct groups. The fact that IMP α3 and IMP α2 are only present in metazoans implies that they emerged during the evolution of multicellular animals to perform specialised roles in particular cells and tissues. This review describes what is known of the IMP α gene family in mouse and in *D. melanogaster*, including a comparitive examination of their mRNA expression profiles in a highly differentiated tissue, the testis. The clear implication of their highly regulated synthesis during the course of spermatogenesis is that the different IMP αs have distinct expression patterns during cellular differentiation, implying tissue/cell type-specific roles.

## INTRODUCTION

In eukaryotic cells, bidirectional transport of molecules between the cytoplasm and nucleus relies on access through the nuclear pore complexes (NPC’s) embedded in the nuclear envelope. The NPCs are large multiprotein structures consisting of over 40 different types of nucleoporin proteins that create a symmetrical pore lined with hydrophobic binding sites [1, 2]. Molecules >40 - 60 kDa cannot passively pass through the nuclear membrane and must be actively transported through the NPCs *via *carrier proteins a major class of these being the *karyopherins* [2]. Karyopherins include members of the importin (IMP) and exportin (EXP) protein families and interact with their protein targets *via *modular Nuclear Localisation Signals (NLSs) or Nuclear Export signals (NESs), respectively, that are encoded within the target cargo protein. NLSs may be either monopartite, consisting of a single cluster of basic amino acids, or bipartite, consisting of 2 such clusters separated by a 10-12 amino acid linker region [3].

The most well characterised mechanism of nuclear import is mediated by the IMP α and IMP β1 heterodimer. Cargoes bind to IMP α, and this complex is targeted by IMP β1 to the NPC where it docks with the aid of the nucleoporins that form the NPC. Upon translocation through the pore, binding of the small GTPase Ran, as Ran-GTP, by IMP β1 potentiates dissociation of the complex and cargo release [1, 2, 4].

The IMP α proteins were the first recognised cytosolic factors required for selective nuclear import of proteins containing an NLS. *In vitro* reconstituted nuclear transport in *Xenopus laevis* oocytes with recombinant IMP α2 was found to be dependent on the combination of an NLS, the presence of Ran and a mechanism for energy regeneration within the experimental system [5]. The *X. laevis* cloned sequence displayed 44% amino acid similarity with yeast Srp1p, an essential protein in* S. cerevisiae* (yeast), which suppressed temperature sensitive RNA polymerase I mutations and was associated with the nuclear pore complex [6]. A yeast-2-hybrid screen with nuclear protein Human Lymphoid Enhancer Factor-1 identified *M. musculus *(mouse) Srp1 (α1) and Pendulin (α2), [7] and these were later used to identify a further 3 IMP α genes in the mouse using degenerate PCR [8], bringing the current total to 5 IMP α isoforms recognised in this species.

Phylogenetic analyses suggest that the IMP αs can be classified into three distinct subfamilies which display approximately 50% amino acid identity between groups and 80% within groups [2, 8, 9]. Complications in nomenclature exist due to the multiple names that have been given to the IMP α family members thus far, and therefore a summary of corresponding names is provided in Table **1** (see also [10]). Nomenclature used in this review will follow the IMP α-*number* type terminology. 

## IMP α STRUCTURE

The IMP α proteins are comprised of 3 main structural domains: 1) the highly-conserved hydrophilic N-terminal region which is the importin β binding (IBB) domain; 2) a hydrophobic central domain containing Armadillo (ARM) motifs; and 3) a non-conserved C-terminal region. The IBB domain mediates IMP β1 binding and thus enables cargoes to be targeted to the NPC for nuclear import [11]. Additionally, it interacts with the NLS binding regions within the ARM motifs, to inhibit cargo binding when not bound to IMP β1 [11-13]. The ARM motifs of the central domain comprise 3 α helices organised into a right-handed superhelix [14]. ARM repeats 2-4 and 7-8 contain binding sites for monopartite and bipartite NLSs.

Substantial evidence demonstrates that cargos display preferential utilisation of particular IMP αs [15-20]. For example, RCC1, which mediates GDP/GTP exchange on Ran, exhibits specificity for IMP α3 in contrast to all other IMP αs (Kohler *et al*., 1999; Quensel *et al*., 2004; Talcott and Moore, 2000). The specificity of cargoes for particular IMP αs appears to rely on structural features of the cargo outside of the NLS region which enhance their binding. For example, in the NLS-exchange between RCC1 and a histone chaperone protein, nucleoplasmin, the NLS alone of RCC1 was unable to confer strong IMP α3 specificity on nucleoplasmin due to the fact that the C-terminis ‘propeller’ domain of RCC1 is responsible for enhancing the interaction with IMP α3 [21].

All of the IMP subfamilies mediate nuclear import (reviewed [22], but they also play roles in additional cellular processes including ubiquitin-mediated protein degradation and mitotic spindle organisation, the latter of which does not appear to be a direct result of an effect on nuclear import [23-25]. At the commencement of mitosis when nuclear envelope breakdown occurs, IMP α1/β complexes bind to and hold inactive spindle assembly factors including TPX2, NUMA and XCTK2. Chromosome-associated RCC1 produces high levels of Ran-GTP in the vicinity of the chromosome; this is proposed to release these spindle assembly factors in close proximity to the chromosomes where they are required [26-28]. IMP α2 has also been implicated in ring canal formation during *D. melanogaster* oogenesis. In null IMP α2^D14^ mutants, cytoplasmic bridges between nurse cells and growing oocytes are disorganised. This has been attributed to mislocalisation of the Kelch protein, which is required for cross-linking of actin filaments during canal formation (Gorjanacz 2002); whether IMP α2 binds directly to Kelch has not been determined, but IMP α2 association with the oocyte cortex and the F-actin cytoskeleton during *D. melanogaster* oogenesis has been demonstrated [29].

## THE IMP α GENE FAMILY

The budding yeast genome contains only a single IMP α gene, *Srp1* [6, 30], whereas *D. melanogaster* has at least 3, mouse, 5, and human, 6. Yeast and plant IMP αs are orthologs of the α-1 family, whilst metazoans contain members of all three subgroups [31]. Phylogenetic analyses of IMP αs across invertebrates, plants, fungi and vertebrates have been presented elsewhere [31]. The expansion of the IMP α gene family during evolution suggests that the different IMP α genes have developed specialized roles to support the development of highly differentiated tissues. In light of this observation, the genomic structure of the IMP α genes of an invertebrate and a vertebrate model species is compared in the current review. *D. melanogaster* contains a single member of each IMP subgroup and thus represents a useful model to elucidate the specific functions of each IMP α subfamily.**That invertebrates contain members of all three key IMP α groups is suggestive of a cellular requirement for this protein family at an evolutionary stage preceding invertebrate-vertebrate divergence [31]. The mouse represents a more complex eukaryotic model which has multiple members of some of the α subgroups. 

At the amino acid level, the *D. melanogaster* IMP αs (dIMP αs) display 40-65% identity to the mouse IMP αs (mIMP αs) (Table **2**). The classification of mIMP α3 and 4 to the same subgroup (α-Q/α-3) family is based on their evident high level of identity (85%), as exists between mIMP α1 and α6, to the α-S/α-1 sub-family, with 81% identity. The dIMP α1 shows highest similarity with mIMP α1 and α6 (61%), dIMP α2 with mIMP α2 (47%), and dIMP α3 with mIMP α3 and 4 (66 and 65% respectively), and phylogenetic analysis indicates that all 3 major subgroups are represented within this invertebrate species. For both species, the higher percentage identity and thus closer homology between the α-P/2 and α-Q/3 subgroup members favours the view that these groups were evolutionarily derived from a pre-α1 [31].

Also of note from an evolutionary viewpoint is that the order of IMP α ARM repeats appears to be conserved from yeast to humans. This has been determined by phylogenetic analysis of each individual ARM repeat from yeast IMP α and human IMP αs 1 and 2 [32]. Thus, for example, ARM repeat 1 of yeast is most similar to ARM repeat 1 in human IMP α1 and in human α2, than to any of the other ARM repeats contained within these proteins. This indicates that order of the ARM repeats and the NLS regions that may be contained within them, is indeed functionally important, and has been maintained through evolution from the progenitor IMP α [32].

**Fig. (1).**
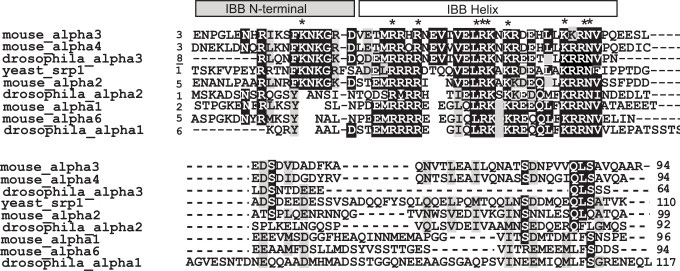
**Alignment of mouse, drosophila and yeast IMP α  IBB domains.**Completely conserved residues are indicated by an asterisk (*).Identical residues at a given position are highlighted in black, similar residues are highlighted in grey.

The IBB domain of the IMP αs displays significant homology across species, particularly the N-terminal component (a.a 11 to 23 of mouse IMP α2) and the helix component of the domain (a.a. 24 - 51) (Fig. **1** and [33]). These two regions lie perpendicular to one another, with residues of the N-terminal region interacting with the HEAT repeats 7-11 of IMP β1 and residues of the IBB helix interacting with the HEAT repeats 12-19 [33]. The HEAT repeats are ~40 residue long tandem repeats that constitute the major part of IMP β structure [33]. The IBB helix component is the most conserved region of the IBB domain displaying 33% identity at the amino acid levels between all yeast, drosophila and mouse IMP αs (Fig. **1**). Most of the conserved residues are basic, thereby allowing critical electrostatic interactions with the acidic inner surface of IMP β1 HEAT repeats [33].

## MOUSE AND DROSOPHILA IMP α GENES: GENOMIC STRUCTURES

The *D. melanogaster* genome contains a single gene encoding IMP αs 1, 2 and 3, each located on different chromosomes. Three transcript variants of IMP α3 arise from alternative promoter usage, all encoding the same polypeptide of 514 amino acids in length (Table **3**). A putative 4^th^ IMP α-like gene, CG10478, also exists, although the protein encoded displays significantly lower similarity to other vertebrate IMPs, exhibiting the highest identity (36%) to mouse IMP α4. CG10478 is most similar to *Drosophila* IMP α3 and may represent a *Drosophila*-specific gene duplication. In contrast, the mouse genome encodes a single gene for each IMP αs 1, 3, 4 and 6, and all map to different chromosomes. 3 pseudogenes also exist for IMP α2, each on different chromosomes. Multiple transcripts exist for all mouse IMP αs except mIMP α4, with these transcripts falling into both the coding and non-coding categories. Only the mIMP α1 and α2 genes appear to produce non-coding transcripts. The different mIMP α2 transcripts products from the Chromosome 11 gene arise as a result of alternative promoter usage and alternative splicing mechanisms, as illustrated in Fig. (**2**).

**Fig. (2).**
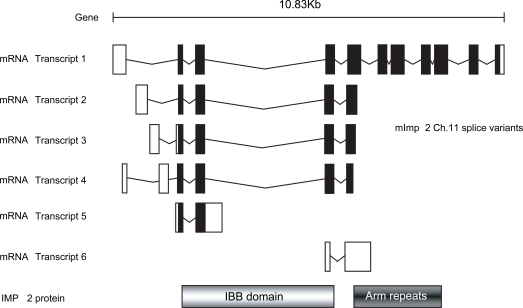
**Summary of the IMP α2 transcripts derived from the chromosome 11 locus**.Mouse IMP α2 yields the most diverse range ofgene products of all the IMP α subfamilies. Six different transcripts are produced from this locus *via* alternative splicing and use of alternative transcriptional start sites, with 5 of these coding for polypeptides. Exons are indicated by black boxes and introns, as lines. Untranslated regions of transcripts are indicated by the white boxes. Regions coding for the IBB domain and ARM repeats are shown, revealing that transcripts 4 and 6 lack the ARM repeats. (Derived from Emsembl(http://www.ensembl.org/ ) Reference OTTMUSG00000003580).

It is notable that two of the mouse IMP α2 coding transcript variants and one of the IMP α6 coding transcripts code for peptides that lack the ARM repeat regions (Fig. **2** and Table **3**). Little investigation into the functional significance of these isoforms has been performed, yet at least one report indicates these truncated forms may be required for normal cellular function. The human breast cancer cell line ZR-75-1s expresses a truncated IMP α2 isoform (1-89aa) which lacks the ARM repeats. The tumour suppressor protein p53 can bind full length IMP α2, but not the truncated isoform which lacks the putative p53 NLS binding domain [34]. *In vitro* p53 is mislocalised to the cytoplasm in cells over-expressing the mutant IMP α2. This suggests truncated IMP α2 may act *in vivo* as an inhibitor of nuclear transport, potentially *via *competition with full length IMP α for IMP β binding [34]. Whether other truncated IMP α transcripts encode proteins that fulfil similar roles and whether this occurs across species, remains to be elucidated.

## DIFFERENTIAL EXPRESSION PATTERNS OF THE IMP αs

The IMP α genes in eukaryotes have been reported as displaying cell and tissue-specific expression patterns. Considered together with the differential NLS binding capacities of the various IMP αs, this argues for distinct nuclear transport roles for each of the different IMP α subgroups and the members thereof.

In *D. melanogaster,* selective expression in IMP α 1, 2, 3 throughout development is evident, with mRNA and protein of all 3 genes highly expressed in early embryos, decreasing to undetectable or low levels in larvae and increasing again at the pupae and adult stages, correlating to the developmental stages when most tissue differentiation is occurring [32, 35, 36]. dIMP α2 and 3 are much more highly expressed in adult females than males, whilst the converse is true for dIMP α1 [36-38]. All three IMP genes are expressed in *D. melanogaster* gonads, with IMP α2 and IMP α1 mRNA selectively enriched in the testis relative to the ovary, whilst IMP α3 mRNA is expressed at similar levels in both ovary and testis [31]. In embryos, both dIMP α1 and dIMP α2 localise to the nucleus at the onset of mitosis, suggesting that dIMP α1 and α2 are required for the import of proteins in mitotically active cells rather than acting generally as a protein importers in all cell types [36, 38].

Several reports have highlighted the tissue and cell type specific expression patterns of the IMP α gene family in different mammalian embryonic and adult tissues [7, 8, 39-41]. The major transcripts of mIMP α3 and 4 appear to be constitutively expressed in several species, whilst mIMP αs 1, 2 and 6 show low to non-detectable levels in a number of tissues [7, 8]. Single predominant transcripts of IMP αs 2, 3, 4, 6 are present in most tissues, with multiple-sized transcripts for IMP α4 and 6 also detected in testis [8]. The ~2kB IMP α6 transcript appears unique to the testis. With the exception of IMP α1, many of the transcript variants derived from EST sources (Table **3**) appear to be expressed at insignificant levels. For example, probes for IMP α2 have been used on Northern blots which encompassed regions contained in all of the potential transcript variants, but only one prominent transcript corresponding to the longest transcript was detected, indicating others may be not be expressed at all in certain tissues or exist only at relatively low levels [7, 8]. 

## TESTIS AS A DIFFERENTIATED TISSUE MODEL FOR REGULATED IMP EXPRESSION

Gametogenesis represents a complex process of cellular differentiation dependent upon highly coordinated stage-specific cues. Spermatogenesis in *D. melanogaster *and in mouse testis gives rise to distinct cell types that have been relatively well characterised in terms of morphology and gene expression. Spermatogenesis encompasses the significant cellular processes of both mitosis and meiosis with considerable morphological transformations occurring as the diploid cell develops into a haploid, motile cell with a highly condensed nucleus [42, 43]. These features make the testis a particularly useful model for studying the involvement of IMP αs in developmentally stage-specific roles, particularly considering that all the IMP α isoforms of both species are expressed in their respective testis [10, 38]. It has been proposed that the regulated expression of nuclear transport factors and specific nuclear proteins during spermatogenesis may mediate the developmental switches that underlie germ cell differentiation [10, 44, 45].

## *D. MELANOGASTER* SPERMATOGENESIS

In the *D. melanogaster *testis, a group of non-dividing somatic cells form a hub at the apical tip of the testis tubule, surrounded by 5-9 germ line stem cells and by somatic stem cells. Asymmetrical cell division of a germ line stem cell yields a primary spermatogonial stem cell which continues to divide until a 16-cell cyst of spermatogonia is present. These undergo meiosis to yield secondary spermatocytes and then ultimately 64 haploid cells mature into spermatozoa [38, 46].

*D. melanogaster* IMP α1 is expressed at low levels in spermatogonial cells and during the spermatocyte growth period but highly in meiotic spermatocytes and in the early phases of spermatid differentiation. In contrast, dIMP α2 is expressed in the somatic and spermatogonial stem cells, and in spermatocytes until meiosis II while dIMP α3 is not found in spermatogonial cells, only weakly in spermatocytes and strongly in post-meiotic spermatids [31, 38]. The mRNA expression of the IMP αs overlaps during meiosis suggesting that they have a common role in meiosis in transporting a diverse range of cargoes, in addition to performing distinct roles as indicated by their specific expression patterns.

The only phenotypes identified for *D. melanogaster* IMP α2 null mutants relate to fertility [31]. IMP α2 is required, but not essential, for male *Drosophila* fertility whereby homozygous mutant males display reduced fertility with a lack of spermatid individualisation resulting in a severe lack of motile sperm. That the *Drosophila *IMPs perform functionally similar roles to one another is indicated by the observation that transgenes of α1 and 3, in addition to α2, can rescue the fertility of homozygous α2 mutant males [31]. Intriguingly, this functional redundancy does not exist to the same degree in oogenesis. IMP α2 females with homozygous mutations in the NLS or CAS/exportin domains of α2 are defective in transport between oocytes and nurse cells and fertility of such females can only be restored by the α2 transgene [29, 31]. 

In contrast, IMP α3 appears to play a more central role generally in development since dIMP α3 mutants die between the 1^st^ and second instar larval phase. However, dIMP α3 has been implicated in the nuclear transport of Germ cell-less (Gcl) which is required for formation of the primordial germ cells [38, 47]. This may be a unique cargo for dIMP α3, as although dIMP α2 can also bind Gcl, it is with much lower affinity [37]. No specific phenotypes associated with gametogenesis have been reported for IMP α3 mutants, and mutants have yet to be derived for dIMP α1.

## MAMMALIAN SPERMATOGENESIS

The differentiation of the mammalian male germ cell from undifferentiated diploid stem cell to differentiated haploid spermatozoa is driven by endocrine as well as paracrine cues, mediated by and derived from the surrounding somatic Sertoli, Leydig and peritubular myoid cells. Spermatogonia undergo mitosis to yield stems cells that remain in the stem cell pool or alternatively multiply and differentiate into spermatogonia that continue on to undergo meiosis I to give rise to tetraploid pachytene spermatocytes. The second meiotic division yields haploid spermatids. Meiosis is associated with high transcriptional activity promoted by histone acetylation, but once histones are replaced by protamines post-meiosis the altered chromosomal architecture results in cessation of transcription. Post-meiotic differentiation involves gross structural changes that transform the round spermatids into elongated spermatids and ultimately the mature spermatozoa is formed [43, 48].

**Fig. (3).**
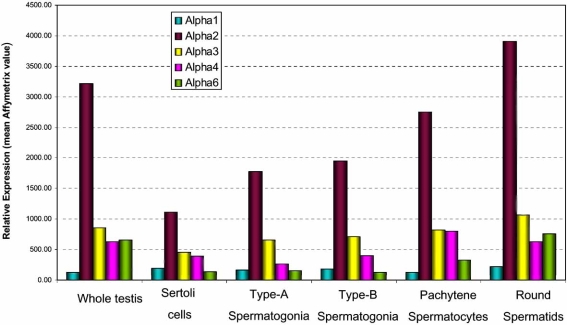
**Microrray-based expression profile of the mouse IMP αs in the developing mouse testis, encompassing the processes of embryonic gonadal development through to spermiogenesis in the adult (NCBI GEO references: GSE2736) [61].**Arbitrary Affymetrix expression values are provided.

Based on previous *in situ *hybridization data [10] and publicly accessible Affymetrix array data summarised and presented herein (Fig. **3**), mouse IMP α1 mRNA exhibits the most ubiquitous expression pattern during spermatogenesis, from the spermatogonium through to the round spermatid stage. In contrast, the remaining IMP αs display more restricted expression patterns. mIMP α2 is highly expressed in the spermatocytes and round spermatids, mIMP α3 is present in spermatogonia and spermatocytes, mIMP α4 is most abundant in spermatocytes, and mIMP α6 in round spermatids (Fig. **3**, and [10]). Thus, IMP α1 and IMP α3 may have distinct roles during mitosis, as well as having overlapping functions with the other IMP α2 and IMP α4 during meiosis. Interestingly, mIMP β1 is not detected past the spermatocyte stage, in contrast to IMP α1, 2 and 6, arguing for roles for these importins in addition to involvement in classical α/β heterodimer nuclear import [10].

**Fig. (4).**
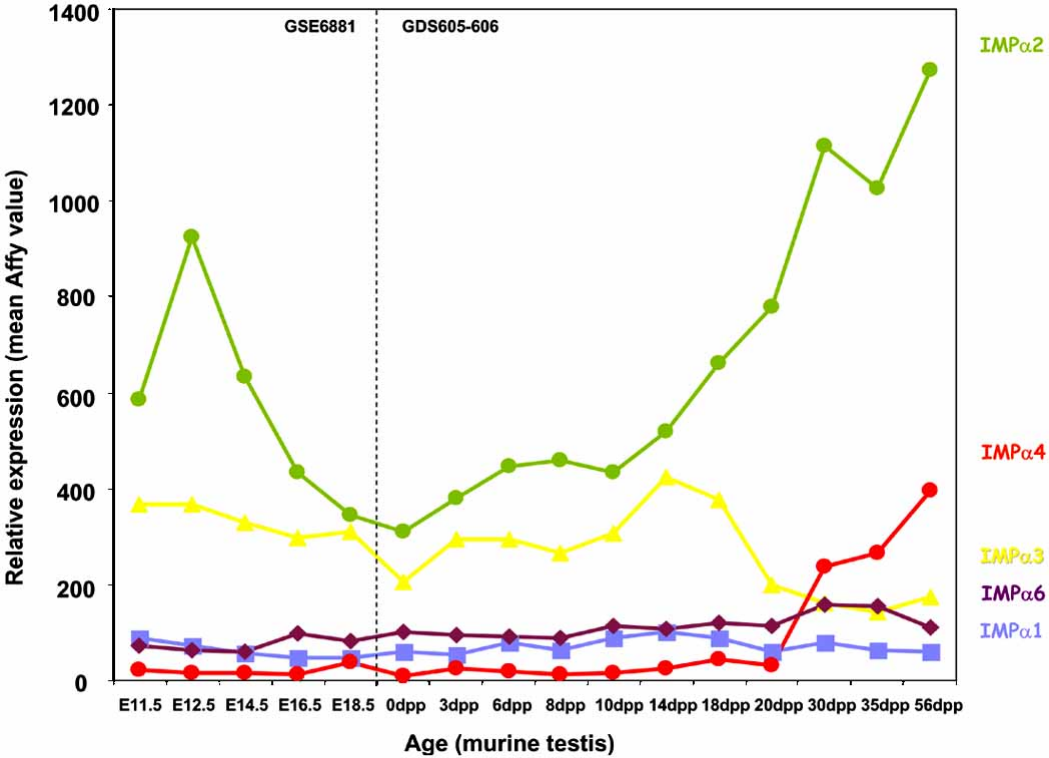
**Microarray based expression profile of the different IMP αs in subpopulations of adult mouse testis cells (NCBI GEO reference:GSE6881, GDS605-606) [57].**Arbitrary Affymetrix expression values are provided.

Consistent with these data, an age series Affymetrix analysis of the IMP αs supports the theory that different importins have distinct functions during development (NCBI reference GSE6881, GDS605-6). The age series examined herein encompasses the period from mouse gonadal differentiation (E11.5-12.5dpp), to adulthood (~56dpp). Throughout this period, distinct gonadal cell types begin to appear at specific time points e.g. at day 0 only quiescent gonocyte are present; by ~10dpp primary spermatocytes exist and by 35dpp mature spermatozoa are present (Fig. **4**). The IMP αs demonstrate distinct expression profiles across this period (Fig. **4**). For example, IMP α2 expression drops dramatically post- gonadal differentiation to ~0 dpp, and rises again at 20 dpp, correlating with when spermatocytes begin to appear in the juvenile testis. IMP α3 expression is maximal at a point where the testis contains only spermatogonia and primary spermatocytes and drops once these are effectively proportionally ‘diluted’ as spermatids and mature spermatozoa develop.

Considering that in both* Drosophila* and* Mus musculus,* members from all three IMP α subfamiles are expressed in the spermatocyte, a conserved role for IMP αs in meiotic processes is indicated, potentially related to spindle assembly. However the distinct expression profile for each of the mouse IMPs also strongly implies unique roles for specific transport or non-transport functions during the more complex process of mammalian spermatogenesis. 

## SIGNIFICANCE OF DIFFERENTIAL EXPRESSION: ROLE OF IMPs IN GENE REGULATION

Integral to controlling access of proteins such as transcription factors to the nucleus, the IMPs play an essential role in gene transcriptional control. However the IMP genes themselves are subject to mechanisms that control their own expression. Regulation of expression of nuclear transport factors alongside regulated expression of nuclear proteins may be a mechanism for control of the specific developmental switches during spermatogenesis [33, 49].

There are many mechanisms that regulate tissue-specific gene transcription. Levels of transcriptional control include DNA accessibility within chromatin that governs transcription factor access, modulation of appropriate transcription factor availability to interact with the corresponding promoters, transcription of the appropriate gene splice variants *via *alternate promoter usage and alternate mRNA splicing and post-transcriptional regulation of mRNA as a means of temporal control of translation. As a means of elucidating upon how the IMP α genes themselves are regulated, we investigated IMP α regulation at the level of transcriptional initiation, by using *in silico* promoter analysis to investigate potential promoter regions of the IMP α genes in the mouse.

## PROMOTER ANALYSIS OF THE IMPORTIN α GENES

The K-SPMM database [50] describes the location of promoters, transcription factor binding sites and the location of transcription factor binding modules of genes expressed during spermatogenesis in the murine testis. We used TRANSFAC analysis to identify additional non-spermatogenic-specific features [51]. The promoter sequences analysed in K-SPMM incorporated 1 kB upstream of the Transcriptional Start Site (TSS) of the most 5’ TSS of splice variants, whilst non-spermatogenic TRANSFAC was extended to 2 kB upstream and ~50 bp downstream of the TSS. The K-SPMM analysis additionally allowed for identification of promoter element modules that are associated with, or excluded from, specific testis cell types: Sertoli cells, spermatogonia, spermatocytes and spermatids.

The promoter modules identified using the K-SPMM/TRANSFAC analysis for a representative member of each of mIMP α subfamily (IMP αs 2, 4 and 6) are given in Figs. (**5**-**7A**)). By way of example, the K-SPMM data obtained also provides some information on conservation within select module regions relative to four other vertebrate genomes (*H. sapiens, R. norvegicus, G. gallus and C. familiaris)* as presented in Figs. (**5**-**7B**))*. *In addition, the testis cell types which express other genes with these same putative promoter modules as the IMP αs, are highlighted in Figs. (**5**-**7C**)). 

**Fig. (5):**
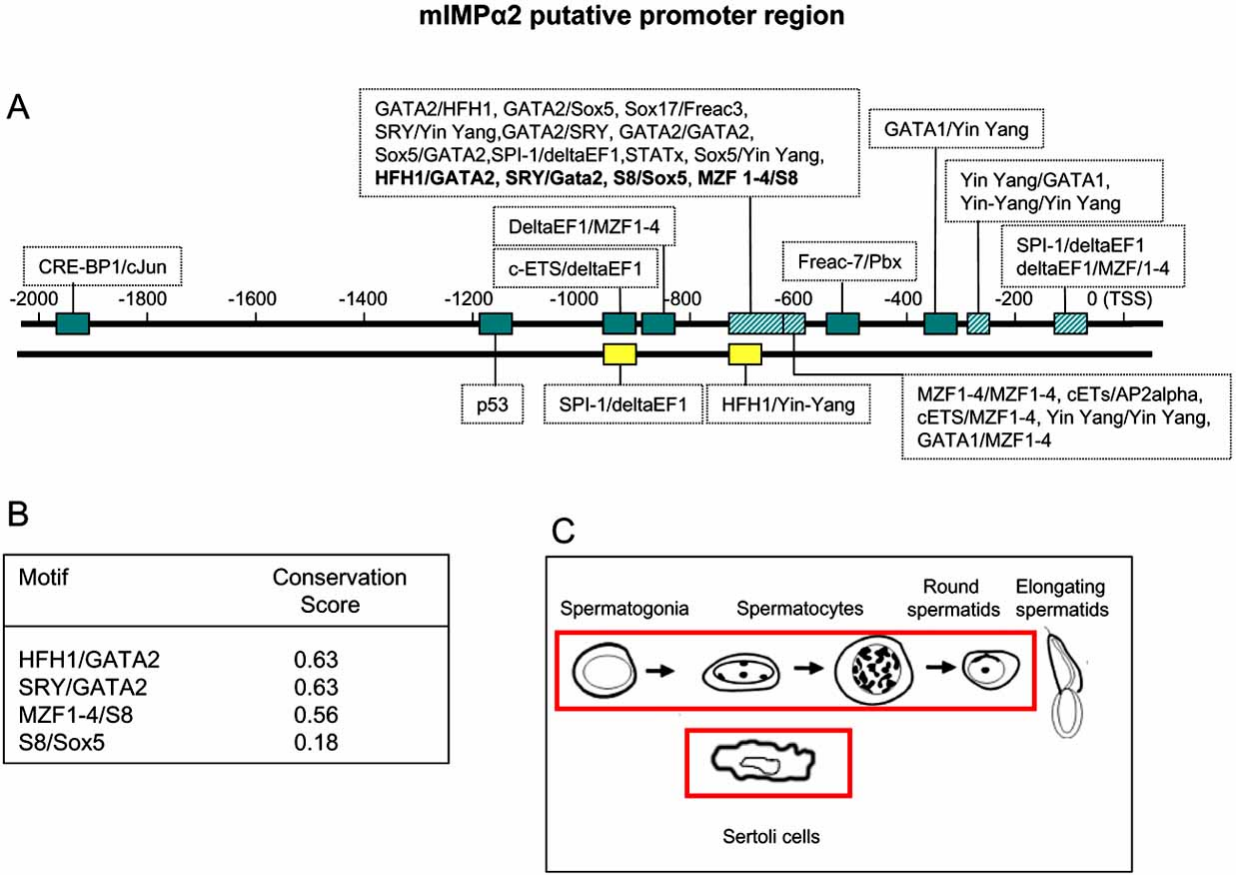
**Figs. (5-7).Identification of paired transcription factor binding sites that are likely to be functional in the putative promoter regions of mouse IMP αs.**A representative member of each IMP α subfamily is given (IMP α2, 3 and 6). Promoter modules 0 to +1κB upstream of the transcriptional start sites (TSS) were identified using the K-SPMM database – a TRANSFAC based, spermatogenesis-specific database. Additional modules (up to +2κB from the TSS) were identified beyond the 1-kB limit of the KSPMM analysis by a TRANSFAC based promoter search not restricted to germ cells (Figs. **4**-**6A**). Those modules displaying particularly high per-base conservation between 4 different eukaryotic species (*M. musculus, H. Sapiens, C. Familiaris, R. Norvegicus and G. Gallus*) are highlighted in bold, and listed with their conservation scores (Figs. 4-6B). The testicular cell types that express other genes with the same promoter motifs (identified *via* K-SPMM) of the given IMP α, are highlighted in Figs. (5-7C). i.e. elongating spermatids do not express mRNAs from genes that possess the motifs detected for importin α2, whereas each of the motifs have been detected in the mRNAs from genes in each of the other testicular cell types.

**Fig. (6):**
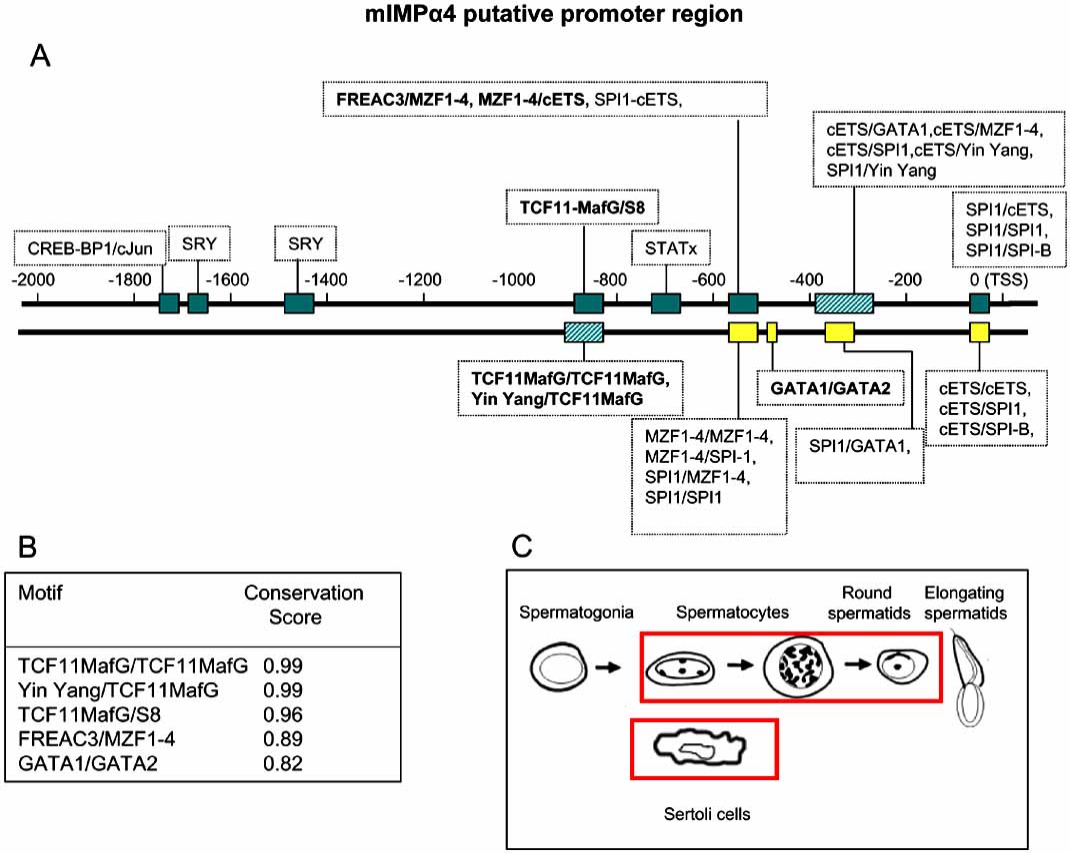
**Figs. (5-7).Identification of paired transcription factor binding sites that are likely to be functional in the putative promoter regions of mouse IMP αs.**A representative member of each IMP α subfamily is given (IMP α2, 3 and 6). Promoter modules 0 to +1κB upstream of the transcriptional start sites (TSS) were identified using the K-SPMM database – a TRANSFAC based, spermatogenesis-specific database. Additional modules (up to +2κB from the TSS) were identified beyond the 1-kB limit of the KSPMM analysis by a TRANSFAC based promoter search not restricted to germ cells (Figs. **4**-**6A**). Those modules displaying particularly high per-base conservation between 4 different eukaryotic species (*M. musculus, H. Sapiens, C. Familiaris, R. Norvegicus and G. Gallus*) are highlighted in bold, and listed with their conservation scores (Figs. 4-6B). The testicular cell types that express other genes with the same promoter motifs (identified *via* K-SPMM) of the given IMP α, are highlighted in Figs. (5-7C). i.e. elongating spermatids do not express mRNAs from genes that possess the motifs detected for importin α2, whereas each of the motifs have been detected in the mRNAs from genes in each of the other testicular cell types.

**Fig. (7):**
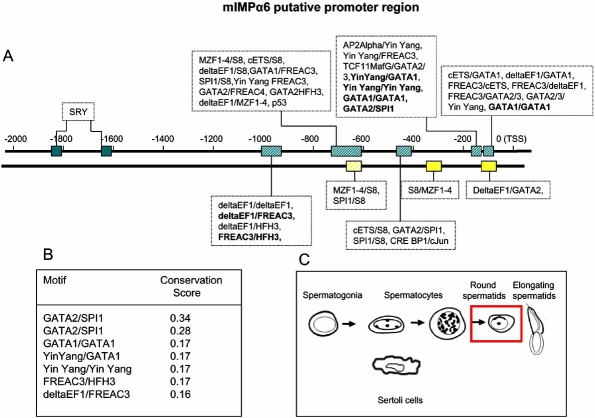
**Figs. (5-7).Identification of paired transcription factor binding sites that are likely to be functional in the putative promoter regions of mouse IMP αs.**A representative member of each IMP α subfamily is given (IMP α2, 3 and 6). Promoter modules 0 to +1κB upstream of the transcriptional start sites (TSS) were identified using the K-SPMM database – a TRANSFAC based, spermatogenesis-specific database. Additional modules (up to +2κB from the TSS) were identified beyond the 1-kB limit of the KSPMM analysis by a TRANSFAC based promoter search not restricted to germ cells (Figs. **4**-**6A**). Those modules displaying particularly high per-base conservation between 4 different eukaryotic species (*M. musculus, H. Sapiens, C. Familiaris, R. Norvegicus and G. Gallus*) are highlighted in bold, and listed with their conservation scores (Figs. 4-6B). The testicular cell types that express other genes with the same promoter motifs (identified *via* K-SPMM) of the given IMP α, are highlighted in Figs. (5-7C). i.e. elongating spermatids do not express mRNAs from genes that possess the motifs detected for importin α2, whereas each of the motifs have been detected in the mRNAs from genes in each of the other testicular cell types.

Amongst those motifs identified that are common to all mouse IMP α putative promoter regions were canonical TATA and GC boxes, as well as motifs for ubiquitously expressed TFs including SPI1, TCF11, cETS1 (Figs. **5**-**7A**). In addition, all mouse IMP αs expressed a variety of binding sites for transcription factors that display restricted patterns of tissue expression, including GATA 1 and 2 and winged helix factor HFH3 [52, 53]. Potential binding sites specific to each of the IMPs were also identified, including FREAC-7 and HFH1 for IMP α2 (Fig. **4**) and FREAC-4 for IMP α6 (Fig. **6A**). Variations in the promoter elements present within the various IMP αs may account for their differential expression patterns. Genes with similar promoter modules represent a class of genes that may be co-expressed at distinct stages of spermatogenesis when the appropriate transcription factors are present. In the human, FREAC-4 expression is restricted to the testis and kidney [54]. Confirmation of expression of transcription factors in the testis that have potential binding sites within the IMP α promoters will be the first step in understanding the regulation of IMP α expression. 

Putative SRY-binding modules were detected in all IMP α promoter regions. In the mouse, SRY expression is restricted to the testis within a very narrow period between 10.5 and 12.5 days post coitum (dpc), the time during which sexual differentiation occurs [55, 56]. Microarray analysis of IMP α levels indicates significant levels of IMP α2 and 3 mRNAs, in particular, are present at 11.5 and 12.5 dpc (Fig. **4**) [57]. Moreover, IMP α2 levels drop dramatically after 12.5 dpc, corresponding to the time when SRY expression abruptly ceases. SRY is transported into the nucleus by IMP β1 and hence it could well play a central role in IMP α gene regulation subsequent to its synthesis [58, 59].

The testicular cell types in which mRNAs from genes which possess these promoter motifs are known to be produced are boxed in red in Figs. (**5**-**7(C)**). Putative mIMP α2 promoter modules are present in other genes of all the spermatogenic cell types, whereas IMP α4 modules are found in all cells except spermatogonia and IMP α6 modules are detected in spermatids only. These *in silico* data do show some correlation with the IMP mRNA expression levels determined *via *microarray and *in situ* hybridisation studies in each of the spermatogenic cells types, with mIMP α2 highly expressed in spermatocytes and spermatids and mIMP α6 predominantly expressed in round spermatids (Figs. **3,4** and [10]). The IMP α4 data correlates with high expression of mRNA in spermatocytes and spermatids which are represented in the putative modules detected (Figs. **3,4** and **5C**). However consideration must be given to the likelihood that not all TFBP regions are identified using KSPMM, and the putative modules detected may not actually be functional in a particular cellular context. These data illustrate that *in silico *data may provide a useful tool for identifying potential transcription factor sites and promoter regions of spermatogenic genes, however they must be biologically validated.

The conservation scores of the selected promoter motifs illustrated in Figs. (**5**-**7(B)**) indicates that there is a particularly high level of conservation at the base-pair level for certain promoter regions. For example the TCF11MafG/TCF11MafG, Yin Yang/ TCF11MafG and TCF11MafG/S8 motifs of IMP α4 are 99% percent conserved between the four mammalian species. Such information will aid in identifying the regions and transcription factors binding them that are conserved across evolution and hence likely to be of critical importance.

These *in silico* data provide a useful basis from which to develop models on regulation of IMP α gene expression. In terms of understanding transcriptional control of IMP α genes, modulation of IMP α gene expression represents only one form of IMP α regulation. Other mechanisms include post-transcriptional and post-translational modifications of the IMPs that alter of affinity and accessibility to target NLSs (reviewed in [60]) and must also be considered when unravelling regulation of IMP α activity and its role in developmental systems in general.

## CONCLUDING REMARKS

The IMP α family has undergone significant expansion and specialisation during evolution such that multiple IMP α isoforms and multiple gene products are present in metazoans. Differential tissue and cell- type expression, combined with the distinct cargo specificities of the IMP α and alternate transcript isoforms is highly suggestive of distinct roles for each of the IMP α genes during developmental processes which require strictly regulated and temporally co-ordinated mechanisms of gene regulation. The testis represents an exemplary model for elucidating specific roles of each of the IMP α isoforms, with stage and cell type- specific expression indicating that the importins may indeed trigger progression through the distinct spermatogenic cell types, by controlling the nuclear access of proteins such as transcription factors. *In silico* analysis of putative promoter regions is a point from which to develop strategies to investigate transcriptional control of this gene family. An understanding of the regulation of the IMP α genes themselves will be central to unravelling developmental processes.

## Figures and Tables

**Table 1.** Summary of Yeast, Drosophila and Mouse IMP α  Gene Names and Corresponding Homologs. Alternative Names Used in the Literature are also Provided (Adapted from [8, 10])

Sub-Family	Yeast	Drosophila	Mouse / Other Names
α-S, α-1	Srp1(Z71465)	α1 (NM_079443)	• α1, Srp1β, Rch2 NpI1, αS1 (NM_008465) •α6, αS2 (NM_008468)
α-P α-2	-	α2, pendulin(NM_057693)	•α2, Srp1α , Rch1, pendulin, αP1 (NM_010655)
α-Q α-3	-	α3 (NM_169295)	•α4, Qip1, αQ1(NM_008467) •α3, αQ2 (NM_008466)

**Table 2.** Level of Amino Acid Identity within and between *M.musculus* and *D. melanogaster* IMP α  Proteins. *D.melanogaster* IMP α  Display Approximately 40-65% Percent Identity at the Amino acid Level to their Murine Counterparts

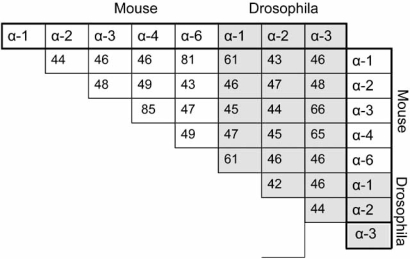

**Table 3. Chromosomal Location and Features of D. melanogaster and Mus musculus IMP α Splice Variants:** Multiple splice variants exist for mouse IMP αs 1,2,3 and 6 and for all Drosophila IMP α  3 only. α2 is the only mouse IMP to be represented by pseudogenes. Truncated mouse transcripts of α2 and α6 that lack the key functional domain of the ARM repeats are indicated. *Drosophila* IMPs αs display less transcript diversity, with the splice variants of Drosophila IMP α3 coding for the same full-length polypeptide. A putative Drosophila α4 gene exists, although this displays relatively low homology (36%) with mouse IMP α4

IMP	Chromosome	No. of Exons	Transcript length (bp)	Protein length (a.a)	Features
**DROSOPHILA**					
**α-1**	3L	6	2307	543	
**α-2** (pendulin)	2L	5	2573	522	
**α-3** Transcript variant A	3R	6	2811	514	
Transcript variant B	3R	6	2779	514	
Transcript variant C	3R	6	2714	514	
**α-4** (putative)	3L	2	1329	442	36%identity with mouse IMP α 4
**MOUSE**					
**α-1**	16	14	4022	538	
	16	14	3211	538	
	16	7	757	145	
	16	3	757	-	Non-coding
	16	3	695	90	
**α-2**	11	11	1,970	529	
	11	5	568	170	
	11	5	659	170	
	11	6	619	129	Lacks armadillo repeats
	11	2	532	80	Lacks armadillo repeats
	11	2	534	-	Non-coding
	4	1	1594		Processed pseudogene
	X	2	1607	-	Processed pseudogene
	2	1	1392	-	Processed pseudogene
**α-3**	14	17	4017	523	
	14	18	1,568	342	
**α-4**	3	17	3726	521	
**α-6**	4	14	5170	536	
	4	4	389	88	Lacks armadillo repeats
	4	14	2195	533	
	4	2	534	-	Non-coding
	4	3	521	-	Non-coding

(Mouse data derived from Emsembl (http://www.ensembl.org/), References ENSMUSG00000022905, OTTMUSG00000003580, ENSMUSG00000021929, ENSMUSG00000027782, OTTMUSG00000009518; Drosophila data from NCBI (http://www.ncbi.nlm.nih.gov/) References NM_079433, NM_057693, NM_169295).

## ABBREVIATIONS

ARM: = Armadillo repeat
IMP: = Importin
IBB: = Importin β binding
NPC: = Nuclear pore complex
NES: = Nuclear export signal
NLS: = Nuclear localisation signal
